# Understanding consumers’ continuous-use intention of crowdsourcing logistics services: Empirical evidence from China

**DOI:** 10.1016/j.heliyon.2024.e29819

**Published:** 2024-04-17

**Authors:** Yu Liu, Meng Shang, Chunjie Jia, Xin-Jean Lim, Ye Ye

**Affiliations:** a School of Flight, Anyang Institute of Technology, Anyang, 455000, China; b Business College, Yeungnam University, Daegu, 423745, South Korea; c School of Business and Economics, Universiti Putra Malaysia, Selangor, Malaysia; d Business School, Shaoguan University, Shaoguan, 512158, Guangdong, China; e International Business School, Krik University, Bangkok, Thailand; f Business School, Sun Yat-Sen University, Shenzhen, 518107, China

**Keywords:** Crowdsourcing logistics, Service quality, Perceived risk, Trust, Continuous-use intention

## Abstract

Crowdsourcing logistics-based O2O (online to offline) has been increasingly implemented to help individuals or merchants tackle down the problem of intra city instant delivery in China. However, since insufficient control is imposed on free couriers, consumers are subjected to certain risks generated by the uneven service quality provided by free couriers, such that the continuous-use intention to adopt crowdsourcing logistics may be affected in an unexpected manner. A sampling survey was carried out in China's first- and second-tier cities, with 292 valid questionnaires collected. On that basis, the corresponding hypotheses were tested using the partial least squares (PLS) method. The findings of this study revealed that trust, perceived value, and satisfaction positively contributed to continuous-use intention, where trust contributed the most. Perceived risk exerted a significant negative effect on continuous use intention. Trust is capable of notably reducing perceived risk. Crowdsourcing logistics service quality is the critical driving variable of perceived value and satisfaction. Perceived risk has a negative moderating effect on satisfaction-continuous-use intention relationship, showing that the higher the perceived risk, the weaker the effect of satisfaction on continuous-use intention. Given perceived risk, a conceptual model was built by integrating e-CSI model (e-Customer Satisfaction Index Model) and PAM-ISC model (Post-acceptance Model of IS Continuance Model). From the integration, the findings of this study are expected to provide decision-making basis for crowdsourcing logistics platforms to help solve the “last mile” delivery problem.

## 1 Introduction

With the surge in demand for demand-based online-to-offline (O2O) services, China's instant logistics industry has experienced explosive growth in recent years, leading to a greater diversity in online shopping options. The integration between information and physical objects, as well as between online and offline channels, has become increasingly seamless. Consumers now expect faster delivery services within the same city to enhance their real-time shopping experience [1]. According to the China Instant Logistics Industry Development Report for 2021 to 2022, the order volume of China's instant delivery industry reached 35.3 billion in 2022, marking a significant year-on-year increase of 20.07 %. Some products require immediate delivery due to their instant attributes, leading to higher expectations for delivery timeliness and service quality. Consequently, the previous three-day delivery timeframe no longer meets consumer demands, driving the rapid expansion of instant logistics services. Instant logistics, characterized by real-time matching of demand and capacity through global scheduling, presents challenges to local transportation capabilities. Different forms of instant logistics have emerged to address varying demands, including crowdsourcing and self-built capacity organization. Solving the "last mile" delivery capacity issue, meeting user experience expectations, and optimizing rider matching for faster order processing and delivery have become primary technical objectives for instant logistics companies [2].

**Fig. 1:**
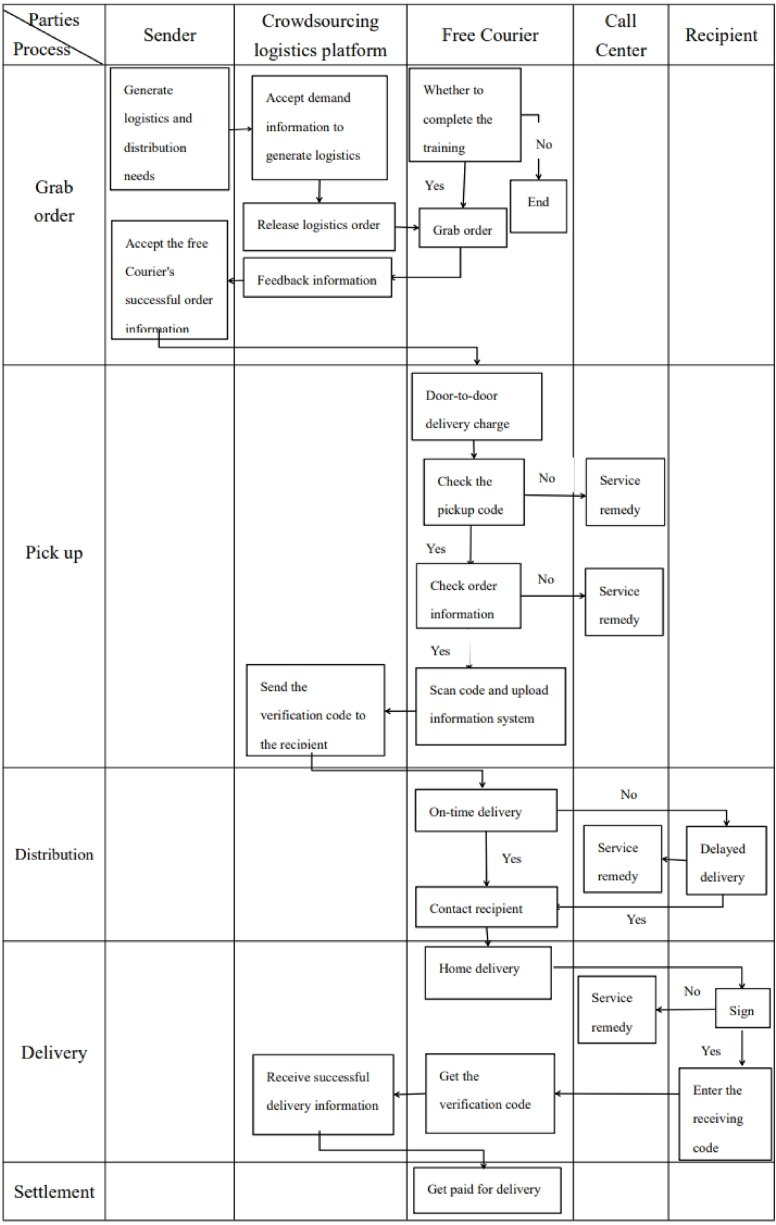
Crowd-sourcing logistics service process.

Given the limitations of traditional logistics models in handling increasing order volumes, a new model based on crowdsourcing has emerged. Crowdsourcing, initially proposed by Jeff Howe in Wired magazine, involves individuals utilizing their free time and resources to address problems or offer opinions without an employment or management relationship with enterprises. This approach leverages loose public resources effectively to assist enterprises in problem-solving. Crowdsourcing logistics employs this model to provide data collection and resource integration services that a single logistics company cannot manage alone. It offers standardized data interaction and business collaboration services to ensure the coordination of logistics, information flow, and capital flow. Utilizing networks or mobile terminals, crowdsourcing logistics matches scattered express delivery demands with available resources efficiently (see Fig. 1).

Due to the discrete nature of upstream orders and their low merging probability within the same city, deliveries often require multipoint-to-multipoint distribution. As a result, crowd-sourcing logistics has emerged as an effective solution to address the timeliness of same-city distribution. Several crowd-sourcing logistics platform companies have arisen, leveraging technical support from mobile online payment and online big data platforms. Renren Express stands as a pioneer in crowd-sourcing logistics in China, with other notable players including JD Daojia, Dada, Shansong, and UUpaotui. Taking Dada Express as an example, it is a leading local instant delivery platform under Dada Group in China, employing crowdsourcing as its core transportation model. Dada Express holds the largest market share in socialized local delivery, covering over 2700 counties, districts, and cities nationwide as of the end of 2020. With a peak daily order volume surpassing 10 million, Dada Express has provided flexible job opportunities for hundreds of thousands of delivery personnel, known as "knights."

Compared to traditional delivery methods, customers demonstrate a heightened level of scrutiny regarding the timeliness, pricing, and reliability of delivery services when utilizing crowdsourcing logistics [3]. Those who engage in crowdsourcing services may also contribute as providers, enhancing the efficiency of social resource allocation. The socialized transport capacity of crowdsourcing logistics effectively meets diverse consumer distribution needs while offering high service flexibility, thus addressing the "last mile" problem [4,5].

However, due to the O2O model upon which crowd-sourcing logistics is built, its physical distribution is managed by independent couriers, whose professionalism varies. As a result, there is inconsistency in the quality of logistics services. Although crowd-sourcing logistics companies reduce labor costs by integrating social idle logistics resources, their control over free couriers is weak due to the absence of formal employment relationships. Consequently, ensuring consistent service quality remains challenging. This results in various issues such as privacy risks, delivery inconsistencies, and performance concerns, impacting users' trust and satisfaction with the service. The lack of transparency regarding the information of crowdsourced couriers also fosters suspicion among users.

As a result, consumer perception of crowdsourcing logistics services directly influences their willingness to continue using these services within an O2O context. Moreover, potential perceived risks further compound this effect, potentially leading to dissatisfaction and discontinuation of service usage. Therefore, as a crowdsourcing logistics platform company, it is crucial to prioritize optimizing the overall service experience, both online and offline, to enhance consumer satisfaction and foster continued usage of crowdsourcing logistics services.

To address these concerns, this study delves into questions regarding how consumers perceive crowdsourcing logistics services, the impact of perceived risk on service continuation, and potential differences in effects between crowdsourcing platforms of varying scales. The subsequent sections of this study will explore these questions further. The organization of this study includes a review of theoretical background and hypothesis development in Section 2, research design in Section 3, empirical results in Section 4, discussion in Section 5, and conclusions and implications in Section 6.

## 2 Theoretical background and hypotheses development

### 2.1 Theoretical background

In this study, we propose an integrative theoretical research model based on the Information System Continuous Use Model (Post-Acceptance Model of IS Continuance, PAM-ISC) and the e-Customer Satisfaction Index Model (e-CSI model). The Expectation Confirmation Theory (ECT) is a prevalent theory in the field of continuous use behavior research [6], primarily employed to explain the correlation between consumers' shopping satisfaction and their intention to continue purchasing. Building upon the Expectation Confirmation Theory, Bhattacherjee (2001) introduced the PAM-ISC model, suggesting that the continuous use of an information system depends to some extent on the user's rational decision-making and their perception of the system's usefulness [7]. Emotional factors also play a significant role in continuous use behavior, including individual user attitudes, satisfaction perceptions, and other emotional-associated factors. This theory has been extensively applied in various contexts, such as online banking [8], web learning [9], and e-government services [10].

The e-CSI model, proposed by Hsu [10], provides another theoretical framework, focusing on the causal relationships between antecedents (customer expectations, perceived service quality, and perceived value) and consequences (customer complaints and customer loyalty) of customer satisfaction [11]. This model emphasizes three key antecedents of customer satisfaction (trust, e-service quality, and perceived value) and two consequences (customer complaints and customer loyalty). Trust replaces customer expectations, and e-service quality replaces service quality, with an additional relationship introduced from trust to customer loyalty. The e-CSI model considers users' perception of e-service quality, emotional responses after service experiences, and their level of trust in service providers or information platforms without face-to-face contact, influencing their perceived emotions after the experience.

Recent research has explored crowdsourcing logistics applications in B2C e-commerce for last-mile delivery [2], customer assessments of e-logistics service quality in crowdsourced delivery [3], evaluation of crowdsourcing modes for parcel delivery service platforms [4], and factors influencing crowdsourcing logistics [5]. However, most empirical studies on crowdsourcing logistics focus on the participation intention of free couriers, with limited research on customer satisfaction and willingness to reuse.

**Fig. 2:**
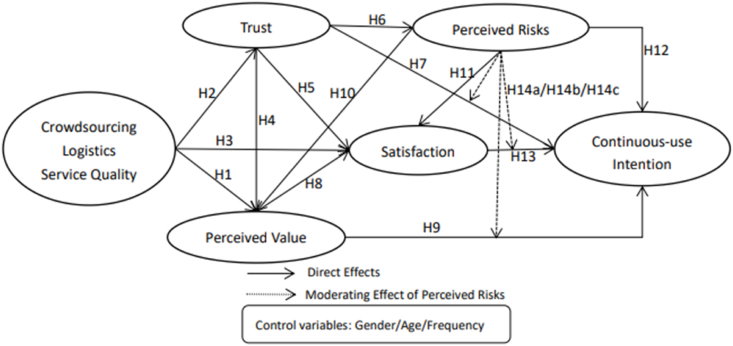
Research model.

Taking into account the novel technological factors within crowdsourcing logistics and the specific characteristics of this model (e.g., involvement of platform parties, merchants, free couriers, and customers), our study integrates elements from the PAM-ISC and e-CSI models. We introduce perceived risk associated with crowdsourcing logistics into our model to explore the structural relationships among antecedents (service quality, trust, perceived value, perceived risk, satisfaction) and consequences (continuous-use intention). The research model, as depicted in Fig. 2, is developed to address these relationships.

Existing research suggests that women tend to perceive a higher level of risk and a lower level of trust in online shopping compared to men [12]. Furthermore, older individuals may prioritize personal value and happiness, while younger individuals may be more attracted to entertainment and fun [13]. Additionally, customers with more online transaction experience may be less concerned about the credibility of the supplier [14]. Consequently, we include age, gender, and frequency as control variables in our proposed research model.

### 2.2 Hypotheses development

#### 2.2.1 Relationships among crowdsourcing logistics service quality and perceived value, trust and satisfaction

Service quality encompasses a holistic assessment of a product or service over the long term, while satisfaction reflects a specific evaluation of a transaction [[15], [16], [17]]. Ahmed et al. [17] identified various dimensions of logistics service quality, including the number of orders processed, packaging quality, delivery timeliness, and reliability, as well as the collaboration between logistics providers and customers [18]. In the context of crowdsourcing logistics, service quality encompasses both online platform interactions and offline delivery experiences. Online service quality pertains to customers' perceptions of electronic service quality (e-SQ), while offline service quality relates to their perceptions of delivery quality [19,20].

According to the e-CSI model, which incorporates e-SQ as an antecedent of trust, perceived value, and satisfaction, electronic service quality can positively influence trust, perceived value, and satisfaction [21]. This is because satisfactory service experiences can foster trust in service providers and enhance customers' perceptions of the value received [22]. Previous research on logistics services has highlighted the role of service quality in enhancing customer trust [17,23] and has established a causal relationship between service quality and customer satisfaction [24,25]. Recent studies have also confirmed the impact of perceived quality and customer satisfaction on intentions to continue using services positively [26].

Therefore, this study proposes the following hypothesis.

H1 Crowdsourcing logistics service quality positively effect on perceived value.

H2 Crowdsourcing logistics service quality positively effect on trust.

H3 Crowdsourcing logistics service quality positively effect on satisfaction.

#### 2.2.2 Relationships among trust, perceived value, satisfaction, perceived risks and continuous-use intention

Trust is crucial in uncertain situations, particularly in the realm of e-commerce, where dominant service platforms operate online, influencing uncertainty mitigation [27]. Consumers tend to trust platforms that they believe will not engage in opportunistic behavior [28]. In the context of crowdsourcing logistics, trust in platforms can alleviate behavioral uncertainty and the associated risk of platform opportunism, impacting consumers' decisions to continue using the platform and recommend it to others [29]. Research indicates that trust directly influences consumers repurchase intentions in the B2C e-commerce sector [29]. Trust in an e-commerce platform can affect consumers' willingness to take the risk of purchasing items sight unseen. Studies have shown that trust in online merchants increases consumers' willingness to shop online and correlates positively with their intention to continue using online services [30].

Trust and risk are closely linked in predicting intentions to use products or services via internet technology. Trust reduces expectations of opportunistic behavior and other uncertainties, thus playing a significant role in reducing perceived risk [31,32]. Additionally, trust enhances consumers' expectations of a product or service's usefulness or performance [33]. Consumers' perceived trust can also influence their overall satisfaction, as trust is a key factor in their assessment of outcomes. Trust not only directly affects loyalty and satisfaction but also indirectly influences them through perceived value [34].

Therefore, trust in crowdsourcing logistics service providers affects consumers' perceptions of value, reduces their perception of risk, increases satisfaction, and enhances their willingness to adopt crowdsourcing logistics services, thereby influencing their participation behavior. Thus, the hypotheses are proposed as follow.

H4 Trust negatively effect on perceived value.

H5 Trust positively effect on satisfaction.

H6 Trust negatively effect on perceived risks.

H7 Trust positively effect on continuous-use intention to adopt crowdsourcing logistics service.

#### 2.2.3 Relationships among perceived value, satisfaction, and continuous-use intention

Perceived value is the consumer's overall assessment of the utility of a product, considering what is received versus what is given, reflecting the trade-off between benefits and costs associated with the product or service. It serves as a relative preference for evaluating performance generated by products, services, and consumption processes [35]. In the context of O2O e-commerce, perceived value represents customers' subjective experiences with crowdsourcing logistics services, ultimately influencing their behavioral decisions. This study proposes structural dimensions of consumers' perceived returns on investment, superior service, enjoyment, and aesthetics.

Existing research highlights the significance of perceived value as a crucial antecedent of overall satisfaction [17]. In the online environment, utilitarian value has been shown to increase customer satisfaction [36], while hedonic value, price, quality, and transactional value are positively correlated with customer satisfaction [37]. Subjective psychological feelings, whether positive or negative, influence consumer satisfaction, which subsequently impacts buying behavior. Studies have demonstrated a positive correlation between perceived value and intention to adopt internet-based services [38,39], with perceived service value serving as a strong predictor of continued intention to use online services [10,40]. Given these findings, it is proposed that.

H8 Perceived value positively effect on satisfaction.

H9 Perceived value positively effect on continuous-use intention.

#### 2.2.4 Relationships among perceived risks, perceived value, satisfaction, and continuous-use intention

Perceived risk encompasses the degree to which an information system user perceives exposure to various risks, including social, security, psychological, financial, physical, performance, or time-related risks [41]. Distrust may arise among parties in virtual communities, with integrity issues among members posing significant challenges [42]. In the context of crowdsourcing projects, risks arise from both online information platform-related factors and offline delivery process-related factors, such as information accuracy, operational performance, distribution security, information release risk, and platform supervision [38,43]. These risks may include concerns about privacy security, delivery reliability, personal and property safety, performance expectations, and legal risks related to compensation.

Research has shown that perceived risks are negatively correlated with the perceived customer value of online shoppers [44], and perceived risk has been found to negatively impact perceived value in various studies [45,46]. Additionally, studies have reported a significant correlation between risk perceptions and negative consumption emotions [47], which directly influence satisfaction and dissatisfaction [48]. Risks associated with online platforms stem from information asymmetry, leading to identity and product uncertainty and fears of opportunistic behavior, thereby reducing transaction intentions [49]. When perceived risk exceeds a user's tolerance level, it can negatively affect their attitude and intention to reuse internet services [50,51]. Furthermore, perceived risk has been shown to significantly influence the intention to use online services [38,52]. Therefore, it is proposed that.

H10 Perceived risk negatively effect on perceived value.

H11 Perceived risk negatively effect on customer satisfaction.

H12 Perceived risk negatively effect on continuous-use intention.

#### 2.2.5 Relationships between satisfaction and continuous-use intention

The Expectation Confirmation Theory is a well-established theoretical framework for studying user satisfaction, positing that users form expectations for a product or service and evaluate it based on their experiences following purchase and use [53]. Satisfaction arises when the actual product or service meets or exceeds the user's expectations, resulting in a feeling of joy [54]. In the context of crowdsourcing logistics, satisfaction occurs when the actual service provided meets or surpasses the user's expectations. Higher satisfaction levels are associated with a greater willingness to make repeat purchases [54], and research has shown that shopping satisfaction positively influences consumers' intention to continue purchasing based on the Expectation Confirmation Theory [2]. Additionally, satisfaction has been identified as a positive factor in the Post-acceptance Model of IS Continuance (PAM-ISC), which explains users' continued use of information systems [55]. Therefore, it is proposed that.

H13 Satisfaction positively effect on continuous-use intention to adopt crowdsourcing logistics service.

#### 2.2.6 The moderating effect of perceived risk

Perceived risk refers to users' subjective belief regarding the potential loss associated with pursuing a desired transaction outcome. When perceived risk surpasses individual tolerance levels, consumers often seek alternatives with lower risk levels to mitigate the negative effects [56]. Highly satisfied customers may express a tendency to switch providers in the presence of perceived risk. Perceived risk may act as a moderator in moderating the relationship between satisfaction and continuous-use intention. It has been suggested that defining perceived risk as a moderator, rather than an antecedent shaping the overall assessment of or satisfaction with services, is more appropriate [57]. Overall, the discussions above suggest that the predictive strength of satisfaction on continuous-use intention may decrease with an increase in perceived risk. Thus, it is proposed that.

H14a Perceived risk has a negative moderating effect on the satisfaction-continuous-use intention relationship.

In an uncertain online environment, consumers may initially consider accepting products or services due to their perceived positive value, but they are likely to reconsider their intention if the perceived risks associated with the purchase exceed their tolerance levels [58]. However, empirical findings from a study on consumers' ride-sharing intentions suggested that perceived risk positively moderates the effect of perceived value on consumers' willingness to use ride-sharing services [38]. Similarly, research on online shopping by Chiu et al. [58] indicated that perceived risk negatively moderates the relationship between utilitarian value and consumers' intention to make repeat purchases, while it positively moderates the relationship between hedonic value and repeat purchase intention [59]. These findings suggest that the impact of perceived value on consumers' repeat purchase intentions may vary under different levels of perceived risk. Moreover, besides directly influencing consumers' continuous-use intentions, an increase in perceived risk may diminish the effect of perceived value on consumers' willingness to continue using crowdsourcing logistics services. Thus, it is proposed that.

H14b Perceived risk has a negative moderating effect on the perceived value-continuous-use intention relationship.

Sahoo et al. [59] proposed that consumers resort to risk-relieving activities to mitigate their perceived risk levels, thereby alleviating feelings of discomfort [60]. These risk-relieving strategies were observed to narrow down the range of alternatives, often favoring well-established brands with solid reputations [61]. Despite having various service options, consumers tend to exhibit more rational decision-making and may opt for alternative products or services due to perceived risks, even if they had previously trusted and used the products or services. Research indicates that the relationship between trust in online shopping and purchase intention can be influenced by perceived risk, with a stronger correlation observed when perceived risk levels are high [62]. Thus, it is proposed that.

H14c Perceived risk has a negative moderating effect on the trust-continuous-use intention relationship.

## 3 Research design

### 3.1 Methodology

The measurement scales utilized in this study were selected based on established literature to ensure the validity and reliability of the measurement instruments. To enhance the effectiveness of the measurement process, professors, researchers, and relevant experts were invited to review the questionnaire. Some items were revised to address inconsistent tenses and grammatical errors. The operational definitions and measurement items of the variables used in this study are detailed in Table 1. The final questionnaire was developed by translating foreign measurement items into Chinese and correcting any misinterpretations. The questionnaire comprised two parts: the first part consisted of 37 questions indicating the measurement variables, while the second part collected demographic information from the respondents, including gender, age, monthly usage, education, monthly income, and occupation.

**Table 1:** Measurement scale.

Construct	Operational Definition	Measurement items	Source References
Crowdsourcing Logistics Service Quality (CLSQ)	The quality perceived by users during all phases of interactions with a crowdsourcing logistics platform, including encounters that occur before, during, and after the transactions	o Accuracy of information transmitted by the crowdsourcing logistics platform oThe speed of information updated by crowdsourcing logistics platform oUsefulness of information provided by crowdsourcing logistics platform oThe ease of operation of the crowdsourcing logistics platform oQuick response and transaction processing capabilities oThe attractiveness of the design of crowdsourcing logistics platform oThe flexibility of distribution by using crowdsourcing logistics oThe service capabilities and attitudes of customer service centers and free delivery companies	oHsu, 2008; Li & Shang, 2020
Perceived Value (PV)	The consumer's overall assessment of the utility of a product, based on perceptions of what is received and what is given	oThe registration and login on crowdsourcing logistics platform is convenient. oThe messages pushed by the crowdsourcing logistics platform can meet customers' requirements. oThe crowdsourcing logistics platform can provide good guide to customers. oThere is completeness of a reasonable refund guarantee mechanism when using crowdsourcing logistics services. oUsing crowdsourcing logistics services can save money. oUsing crowdsourcing logistics services can save time. oUsing crowdsourcing logistics services can save energy. oUsing crowdsourcing logistics services is comfortable. oUsing crowdsourcing logistics services is happy. oUsing crowdsourcing logistics services is interesting.	oWang et al., 2019
Trust(TR)	Users' expectation that the platform behaves ethically, dependably and fulfill their expected commitments	oThe trustworthy degree of the crowdsourcing logistics service platform oThe degree of crowdsourcing logistics platform to keeps promises and commitments oConfidence that crowdsourcing logistics platform has customers' best interests in mind	oGefen, 2000
Satisfaction (SAT)	A cumulative assessment of a firm's offerings about crowdsourcing logistics services	oIn general, I satisfy with the experience of crowdsourcing logistics services oI satisfy with crowdsourcing logistics services compared with expectations oI satisfy with crowdsourcing logistics services compared with traditional distribution	oBhattacherjee, 2001
Perceived Risks (PR)	Users' subjective belief of suffering a loss in using crowd- sourcing logistics	oThe possibility of not to be delivered on time due to bad weather oThe possibility of item damage when using crowdsourcing logistics for distribution oThe possibility of the normal distribution being affected by not updating the information in time oThe possibility of being worried about personal safety when receiving packages from a stranger oThe possibility that customer's private information (name, phone, address, etc.) may be disclosed oThe possibility that a customer may have difficulty in returning an order oThe possibility that when a customer defends his rights, it will be difficult to claim compensation oThe possibility of not good service attitude when using crowdsourcing logistics for distribution oThe possibility of misdelivery of goods due to the uneven qualifications of free couriers	oWang et al., 2019; Hong (2017)
Continuous-use Intention (CUI)	Users' intention to continue to use crowdsourcing logistics	oIntentions to continue using crowdsourcing logistics services rather than discontinue its use oIntentions to continue using crowdsourcing logistics services than use any alternative means oIf possible, the intentions to stop using crowdsourcing logistics services oWillingness to recommend to friends and relatives to use crowdsourcing logistics	oVatanasombut et al., 2008

In variable measurement, crowdsourcing logistics service quality was assessed using a total of 8 items, following the scale of Hsu [10] and Li and Shang [9]. Perceived value was evaluated using a total of 10 items, based on the scale of Wang et al. [37]. Trust was measured with a total of three items, following the scale of Gefen [27]. Satisfaction was assessed with three items, in line with the scale of Bhattacherjee [6]. Perceived risks were examined using a total of nine items, following the scales of Wang et al. [37] and Hong [42], focusing mainly on delivery risk, privacy security risk, law risk, and performance risk. Continuous-use intention was gauged using four items, according to the scale of Vatanasombut et al. [7]. Additionally, control variables such as age, gender, and frequency were included. Respondents used a Likert seven-level scale to indicate their agreement or disagreement with each statement, with 1 = strongly disagree, 2 = disagree, 3 = slightly disagree, 4 = neutral, 5 = slightly agree, 6 = agree, and 7 = strongly agree [63].

To ensure the absence of ethical issues in the questionnaire, the opinion of the school's scientific ethics committee was sought, and the following measures were implemented in the questionnaire survey: First, the questionnaire was anonymous; Second, the content and purpose of the survey were explained to the respondents; Third, participants were given the option to freely answer the questionnaire or not; Forth, the questionnaire did not request personal or private information; Fifth, the data collected in the survey would only be used for this study; Lastly, all participants would receive an online gift after completing the questionnaire.

The data analysis in this study utilized principal component analysis and dimension reduction techniques to determine the measurement variables of the proposed constructs and uncover the internal structure of multiple variables. Following the examination of reliability and validity of the data, structural equation modeling (SEM) was employed, with the partial least squares (PLS) method used through Smart PLS 3.0 software, to assess whether the hypotheses proposed in the conceptual model are supported or rejected.

PLS-SEM was chosen due to its ability to function similarly to multiple regression analysis, focusing on individual path coefficients and variance explained rather than overall model fit. This characteristic makes PLS-SEM particularly suitable for exploratory research purposes. As this study aimed to explore the correlation between service quality, perceived risk, and other attitude variables (such as trust, perceived value, and satisfaction), and their effects on continuous-use intention to adopt crowdsourcing logistics services, the PLS-SEM method was deemed.

### 3.2 Data collection

Given the varying maturity levels of online services across regions with different developmental stages, as well as differences in the acceptance of crowdsourcing logistics, consumers' perceptions of risk and trust in online service platforms can differ significantly. This variance can impact the interpretability of the research model. Therefore, the study opted to select individual consumers who have utilized crowdsourced logistics services in more developed first and second-tier cities or provincial capitals as the research subjects.

For empirical analysis, data were obtained through the distribution and collection of first-hand questionnaires online. The study population comprised individuals or merchants who have utilized crowdsourcing logistics services in China. Provincial capital cities with similar developmental levels were chosen (e.g., Beijing, Shanghai, Hangzhou, Chengdu, Zhengzhou, Xi'an) to mitigate the effects of social and income disparities between developed and underdeveloped areas.

Respondents were asked to indicate their level of agreement with statements related to the respective constructs. An anonymous online survey was conducted over a period of 2 months in 2020, during which a total of 400 questionnaires were distributed. Ultimately, 316 questionnaires were collected, resulting in a recovery rate of 79 %. Of these, 292 questionnaires were deemed valid, yielding an effective rate of 92.4 %.

## 4 Analysis result

### 4.1 Demographic characteristics

Table 2 presents detailed descriptive statistics regarding the demographic characteristics of the respondents. The sample consisted of 151 male respondents (51.7 %) and 141 female respondents (48.3 %). Among them, 183 individuals (62.6 %) were below the age of 30, while 96 individuals (32.9 %) were aged between 31 and 40, and 13 individuals (4.5 %) were above 40 years old, indicating a relatively young sample. In terms of frequency of utilizing crowdsourcing logistics services, 60.2 % of respondents reported using the service once or twice per month, while 39.8 % reported using it more than three times per month.

**Table 2:** Characteristics of the respondents (n = 292).

Characteristic	Frequency	%	Characteristic	Frequency	%
Gender			Education		
Male	151	51.7	High school and below	38	13.0
Female	141	48.3	College	71	24.3
			Undergraduate	153	52.4
			Mater	26	8.9
			Doctor and above	4	1.4
Age			Monthly Income (RMB)		
20 and under 20	40	13.7	Below 5000	156	53.4
21-30	143	48.9	5000∼10000	62	21.2
31-40	96	32.9	10000∼15000	44	15.1
41-50	11	3.8	15000∼20000	19	6.5
Above 50	2	0.7	Above 20000	11	3.8
Frequency (monthly use)			Occupation		
Below 3 times	176	60.2	Students	90	30.8
4–6 times	34	11.6	Corporate/Company Staff	112	38.4
7–10 times	24	8.2	Institutions/Government Staff	32	11.0
Above 10 times	58	20.0	Self-employed/Freelance	40	13.6
			Others	18	6.2

Regarding education level, respondents included individuals with undergraduate education (153 people), college students (71 people), those with a high school education or below (38 people), and graduate-level education (30 people), representing 52.4 %, 24.3 %, 13.0 %, and 10.3 % of the sample, respectively. In terms of monthly income, 53.4 % of respondents earned below 5000 RMB, while 46.6 % earned above 5000 RMB per month. Occupationally, respondents included students (90 people), corporate/company staff (112 people), institutions/government staff (32 people), self-employed/freelancers (40 people), and others (18 people), accounting for 30.8 %, 38.4 %, 11.0 %, 13.7 %, and 6.2 % of the sample, respectively. The sample demonstrated good representativeness and provided insights into the typical situation of crowdsourcing logistics in China's first- and second-tier cities.

To mitigate nonresponse bias, a homogeneity of variance test was conducted for the first 20 and last 20 respondents who completed the questionnaire [64]. The results indicated that all demographic items showed no statistically significant differences between the two groups at the 0.05 probability level, ensuring the reliability of the findings.

### 4.2 Common method bias

To ensure the accuracy of the statistics, it is essential to assess the potential artificial covariation between predictive variables and calibration variables resulting from shared data or respondents, the measurement environment, project context, or project characteristics. Common statistical analyses were employed for this purpose. Harman's single-factor analysis is a widely accepted method for estimating the presence of common method bias in social science research [65]. According to Harman's single-factor analysis, survey data are considered to be less affected by common method bias if the variance explained by a single factor is less than 40 %. Podsakoff et al. [65] similarly suggested that a single factor should be extracted [66]. In this study, Harman's single-factor analysis was conducted, revealing that the proportion of extracted variance was 28.408 %, which is below the threshold of 40 %. This indicates that common method bias was not a significant concern in this study.

### 4.3 Reliability and validity analysis

Confirmatory factor analysis was conducted to assess the reliability and validity of the respective constructs. Table 3 presents the results of the reliability and validity analysis of the primary factors. The Cronbach's Alpha (CA) coefficients for all potential variables exceeded 0.7, and the composite reliability values were all over 0.8, indicating high reliability [67]. Additionally, the average variance extracted (AVE) for each construct exceeded 0.50, and all factor loading values exceeded 0.60 [68], except for CLSQ8, PV1, and PV6, confirming convergent validity. Discriminant validity analysis was performed using both Fornell-Larcher Criteria and Heterotrait-Monotrait Ratio Criteria (refer to Table 4 and Table 5). According to the Fornell-Larcher Criteria, the square root of the AVE for each construct was greater than the correlation values between any two constructs [69]. Regarding the Heterotrait-Monotrait Ratio Criteria, all HTMT values were below the threshold of 0.85 [70], indicating high discriminant validity. To assess the unique explanatory power of a set of independent variables, it is crucial to address the issue of multicollinearity. Variance inflation factors (VIF) were utilized to examine potential multicollinearity problems, with all VIF values found to be less than 5, suggesting no significant multicollinearity issues among the constructs (refer to Table 6) [71].

**Table 3:** Reliability and construct validity.

Construct	Items	Mean	S.D.	Factor Loading	CA	CR	AVE
Crowdsourcing Logistics Service Quality (CLSQ)	CLSQ1	4.68	0.820	0.720	0.908	0.926	0.612
	CLSQ2	4.62	0.875	0.802			
	CLSQ3	4.65	0.906	0.790			
	CLSQ4	4.78	0.848	0.837			
	CLSQ5	4.74	0.850	0.827			
	CLSQ6	4.60	0.813	0.743			
	CLSQ7	4.89	0.712	0.648			
	CLSQ8	4.74	0.825	0.545			
Perceived Value (PV)	PV1	5.20	1.111	0.689	0.906	0.922	0.544
	PV2	5.03	1.061	0.741			
	PV3	5.11	1.075	0.723			
	PV4	5.18	1.124	0.705			
	PV5	5.14	1.078	0.685			
	PV6	4.92	0.827	0.481			
	PV7	5.32	1.051	0.637			
	PV8	5.26	1.110	0.656			
	PV9	5.28	1.089	0.630			
	PV10	5.10	1.119	0.683			
Perceived Risks (PR)	PR1	2.87	1.108	0.717	0.889	0.910	0.530
	PR2	3.08	1.064	0.659			
	PR3	2.97	1.080	0.775			
	PR4	2.95	1.131	0.719			
	PR5	2.81	1.156	0.659			
	PR6	3.08	1.052	0.691			
	PR7	2.99	1.092	0.734			
	PR8	3.01	1.100	0.658			
	PR9	3.05	1.089	0.693			
Trust (TR)	TR1	4.9	1.004	0.729	0.773	0.868	0.688
	TR2	4.98	0.940	0.778			
	TR3	4.85	1.036	0.714			
Satisfaction (SAT)	SAT1	5.67	0.805	0.719	0.723	0.844	0.643
	SAT2	5.64	0.722	0.623			
	SAT3	5.58	0.745	0.695			
Continuous-use Intention(CUI)	CUI1	5.83	1.172	0.600	0.779	0.858	0.602
	CUI2	5.56	1.305	0.710			
	CUI3	5.83	1.090	0.690			
	CUI4	4.62	0.843	0.629			

Note: All factor loadings are significant at 0.001.; CA: Cronbach's alpha; CR: Composite reliability; AVE: Averge variance extracted.

**Table 4:** Discriminant validity (fornell-larcher criteria).

Construct	CLSQ	PV	PR	TR	SAT	CUI
CLSQ	**0.782**					
PV	0.554	**0.737**				
PR	−0.180	−0.450	**0.728**			
TR	0.306	0.449	−0.216	**0.829**		
SAT	0.475	0.496	−0.206	0.411	**0.802**	
CUI	0.357	0.512	−0.424	0.485	0.446	**0.776**

Note: Square root of average variance extracted (AVE) is shown on the diagonal and in bold.

**Table 5:** Discriminant validity (heterotrait-monotrait ratio criteria).

Construct	CLSQ	PV	PR	TR	SAT	CUI
CLSQ						
PV	0.602					
PR	0.197	0.496				
TR	0.359	0.534	0.253			
SAT	0.582	0.599	0.246	0.541		
CUI	0.419	0.607	0.502	0.617	0.585

**Table 6:** Collinearity assessment of constructs.

Construct	CLSQ	PV	PR	TR	SAT	VIF>5?
PV	1.120					NO
PR		1.064				NO
TR	1.000	1.137	1.000			NO
SAT	1.464	1.954	1.262	1.261		NO
CUI		1.744	1.253	1.353	1.424	NO

### 4.4 Hypotheses testing

The hypothesis test for the research model presented in this study was conducted through bootstrapping to assess the significance between the paths of the structural model. Fig. 2 and Table 7 illustrate the results of the structural equation model. The analysis revealed that satisfaction significantly contributed to continuous-use intention (β = 0.191, t-value = 2.634, p < 0.001), thus confirming H1. Trust positively and significantly impacted continuous-use intention (β = 0.275, t-value = 4.301, p < 0.001), perceived value (β = 0.251, t-value = 4.200, p < 0.001), and satisfaction (β = 0.216, t-value = 3.215, p < 0.001), while negatively affecting perceived risks (β = −0.216, t-value = 3.431, p < 0.001). Consequently, H2, H3, H4, and H5 were supported. Perceived risks exerted a significant negative effect on perceived value (β = −0.320, t-value = 4.987, p < 0.001) and continuous-use intention (β = −0.240, t-value = 4.229, p < 0.001), but did not significantly affect satisfaction (β = 0.002, t-value = 0.037, p > 0.05). Therefore, H6 and H8 were supported, while H7 was rejected. Crowdsourcing logistics service quality had a significant positive effect on perceived value (β = 0.419, t-value = 5.877, p < 0.001), trust (β = 0.306, t-value = 4.253, p < 0.001), and satisfaction (β = 0.271, t-value = 2.331, p < 0.05), confirming H9, H10, and H11. Perceived value positively and significantly affected satisfaction (β = 0.250, t-value = 2.505, p < 0.05) and continuous-use intention (β = 0.187, t-value = 2.112, p < 0.05), thus supporting H12 and H13.

**Table 7:** Analysis of the results.

Hypothesis	Path	Path Coefficients	VIF	S.D.	T-Value	P-Value
H1	SAT- > CUI	0.191**	1.424	0.073	2.634	0.008
H2	TR - > CUI	0.275***	1.353	0.064	4.301	<0.001
H3	TR - > PR	−0.216***	1.000	0.063	3.431	0.001
H4	TR - > PV	0.251***	1.137	0.060	4.200	<0.001
H5	TR - > SAT	0.216***	1.261	0.067	3.215	0.001
H6	PR - > PV	−0.320***	1.064	0.064	4.987	<0.001
H7	PR - > SAT	0.002	1.262	0.054	0.037	0.971
H8	PR - > CUI	−0.240***	1.253	0.057	4.229	<0.001
H9	CLSQ - > PV	0.419***	1.120	0.071	5.877	<0.001
H10	CLSQ - > TR	0.306***	1.000	0.071	4.253	<0.001
H11	CLSQ - > SAT	0.271*	1.464	0.116	2.331	0.02
H12	PV - > SAT	0.250*	1.954	0.100	2.505	0.012
H13	PV- > CUI	0.187*	1.744	0.089	2.112	0.035
Control Variables	Age - > CUI	−0.029		0.044	0.659	0.510
	Gender - > CUI	−0.013		0.052	0.258	0.796
	Frequency - > CUI	0.082		0.048	1.704	0.089

Note:*p < 0.05; **p < 0.01; ***p < 0.001.

**Fig. 3:**
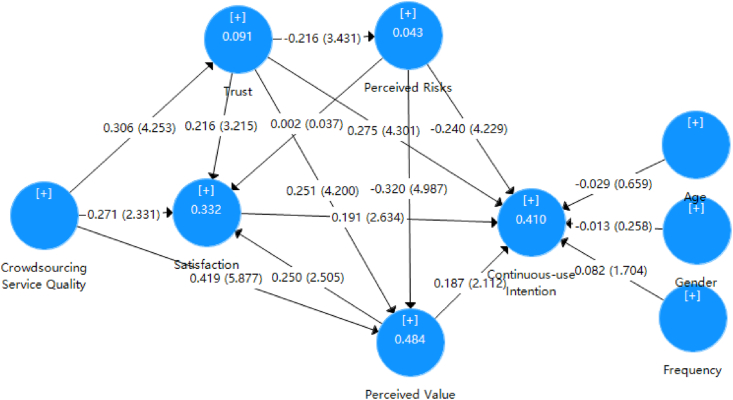
Results of Structural model.

Among the determinants of continuous-use intention (R2 = 0.410), trust significantly influenced continuous-use intention more than perceived value and satisfaction. Among the determinants of satisfaction (R2 = 0.332), crowdsourcing logistics service quality played a more significant role in satisfaction than perceived value and trust. Among the three antecedents of perceived value (R2 = 0.484), crowdsourcing logistics service quality had a more pronounced effect on perceived value than trust (see Fig. 3). As for the control variables, age, gender, and the frequency of using crowdsourcing logistics did not exert any significant effect on continuous-use intention.

### 4.5 Moderating effect of perceived risk

As per the test results for the main effects mentioned above, including the satisfaction-continuous-use intention relationship, the trust-continuous-use intention relationship, and the perceived value-continuous-use intention relationship, along with the significant negative impact of perceived risk on continuous-use intention, PLS-SEM was utilized via bootstrapping calculation to investigate the moderating effect of perceived risk.

The findings indicate the significance of the correlation between the interactive item SAT*PR and CUI (β = −0.204, t-value = 2.126, p < 0.05), supporting H14a. This suggests that perceived risk negatively and significantly moderates the relationship between satisfaction and continuous-use intention. Consequently, higher perceived risk weakens the correlation between customer satisfaction and continuous-use intention, while lower perceived risk strengthens it. These results are consistent with previous findings from Yuksel and Yuksel [72] and Cho and Lee [73].

Similarly, the moderating effect of perceived risk on the perceived value-continuous-use intention relationship was tested. However, the results indicate that hypothesis H14b is not supported, as the interactive item PV*PR has no significant influence on CUI (β = −0.052, t-value = 1.088, p > 0.05). This suggests that consumers' continuous-use intention, generated by perceived value, remains unaffected by the level of perceived risk. Even in the presence of perceived risk, consumers' perception that the service is worth using again remains unchanged. These findings are consistent with the results observed by Chang and Chao [75].

Furthermore, the moderating effect of perceived risk on the trust-continuous-use intention relationship was examined, revealing no significance of the correlation between the interactive item TR*PR and CUI (β = −0.107, t-value = 1.657, p > 0.05). Consequently, H14c is rejected. This indicates that regardless of whether the level of risk perception is high or low, there is no significant effect on the correlation between trust and continuous-use intention. This differs from the research results reported by Qalati et al. [62] One possible explanation is that when consumers trust a service provider, the negative effect of perceived risk is mitigated, and consumers become less sensitive to risk, believing that the service provider will ensure sufficient security to prevent losses, thereby maintaining their intention to reuse the service.

## 5 Discussion

The results of this study strongly support the proposed conceptual model. By exploring the influencing variables of continuous-use intention and the interactions between them, a better understanding of the dependent variable continuous-use intention is achieved. Among the four antecedents directly affecting continuous-use intention, trust emerges as the most significant factor. Satisfaction and perceived value have direct effects on continuous-use intention, while they are influenced by trust and perceived risk. Trust plays a crucial role in promoting satisfaction and perceived value, while also reducing perceived risk, underscoring its critical significance in the model. Trust not only directly facilitates continuous-use intention but also enhances it by optimizing the intermediate variables of perceived value and satisfaction. Additionally, higher levels of trust correspond to lower levels of perceived risk, mitigating the decline in continuous-use intention associated with high perceived risk.

The inclusion of perceived risk in the model proves effective, as it exerts direct negative effects on perceived value and continuous-use intention. Higher perceived risk levels correspond to lower perceived value and continuous-use intention, while reducing perceived risk can effectively enhance perceived value and continuous-use intention. Although the direct correlation between perceived risk and satisfaction was not verified, contrary to previous research conclusions, perceived risk significantly moderates the correlation between satisfaction and continuous-use intention. This suggests that as perceived risk decreases, the correlation between satisfaction and continuous-use intention strengthens, and vice versa. Therefore, building high trust and low-risk service perceptions among users is crucial for promoting the reuse of crowdsourcing logistics services.

While the moderating effect of perceived risk on the correlation between trust and continuous-use intention and the correlation between perceived value and continuous-use intention was not verified, contrary to previous research, the findings shed light on the current attitudes of Chinese consumers towards crowdsourcing logistics services and their willingness to reuse them. Trust, perceived value, and satisfaction positively affect continuous-use intention and serve as post variables of crowdsourcing logistics service quality. Notably, service quality has the greatest impact on perceived value, surpassing its effects on trust and satisfaction. Strengthening the service quality of O2O-based online crowdsourcing platforms, customer service centers, and offline free couriers can enhance trust in crowdsourcing logistics service providers, improve the perceived benefits of users, and ultimately promote satisfaction and continuous-use intention.

## 6 Implications of the study

### *6.1* Theoretical implications

This article represents an effort to establish a structural framework linking service quality, perceived value, trust, perceived risks, satisfaction, and continuous-use intention within the emerging domain of crowd-sourcing logistics. Given the multifaceted nature of crowd-sourcing logistics, which involves various stakeholders, including platform operators, merchants, free couriers, and customers, understanding the structural relationships among these variables is crucial.

One notable aspect of crowd-sourcing logistics is the limited control over free couriers engaged in terminal distribution, which introduces significant risk factors. The article delves into the moderating effect of perceived risks in crowd-sourcing logistics on key relationships, such as those between satisfaction and continuous-use intention, perceived value and continuous-use intention, and trust and continuous-use intention. By doing so, it aims to provide valuable insights into managing customer satisfaction and promoting continued usage within the crowd-sourcing logistics industry.

While the study does not introduce new variables, it leverages existing constructs from established models like the Post-acceptance Model of IS Continuance (PAM-ISC) and the e-Customer Satisfaction Index Model (e-CSI). However, it adapts these models to the unique context of crowd-sourcing logistics, considering the novel technological factors and the diverse stakeholder dynamics involved.

By elucidating the structural relationships among these factors, the article sheds light on critical management considerations related to customer satisfaction and continuous usage in crowd-sourcing logistics. This understanding can inform strategic decisions and operational practices aimed at improving service quality, mitigating risks, and fostering trust among stakeholders, ultimately contributing to the sustainable growth and success of crowd-sourcing logistics ventures.

### 6.2 Managerial implications

The conclusions drawn from the study offer valuable insights for managing crowd-sourcing logistics services effectively. Firstly, it is crucial for providers to prioritize improvements in service quality, encompassing aspects such as information accuracy, service center responsiveness, and delivery quality by free couriers. By focusing on both online and offline service quality management, providers can ensure a seamless customer experience, meet expectations, and ultimately enhance satisfaction, thereby encouraging continuous service usage. Secondly, building and reinforcing consumer trust in the crowd-sourcing logistics platform is essential. This can be achieved through establishing a strong brand image, fostering user engagement, and enhancing transparency in the service process. Strengthening consumer trust not only mitigates perceived risks associated with the service but also increases perceived value and satisfaction, ultimately driving continued usage. Finally, proactive management of potential risks is imperative to ensure the sustainability of crowd-sourcing logistics services. Providers should implement robust management systems to enforce constraints and incentives for couriers, offer comprehensive training programs, and establish mechanisms for tracing the delivery process. By mitigating various risks, such as distribution, privacy security, legal, and performance risks, providers can enhance continuous-use intention and strengthen the correlation between satisfaction and continued service usage. These management strategies can optimize resource utilization, reduce costs, meet diverse customer needs efficiently, and address challenges associated with "last mile" delivery. Overall, implementing these recommendations can help crowd-sourcing logistics platforms improve operational efficiency, build stronger customer relationships, and achieve long-term success in the marketplace.

## 7 Conclusion and future research directions

Over the past few years, O2O-based crowdsourcing logistics services have emerged as a popular solution for enhancing delivery capabilities and meeting customers' demands for faster, more diverse, and more personalized "last mile" delivery experiences. However, inherent risks such as limited control over free couriers and inconsistent delivery quality in crowdsourcing logistics services pose challenges that may lead to dissatisfactory experiences, ultimately affecting consumers' continuous-use intention. In this study, a research model was proposed by integrating the characteristics of crowdsourcing logistics with established models such as e-CSI and PAM-ISC, and introducing perceived risk to investigate the structural relationships between service quality, perceived risk, trust, perceived value, satisfaction, and continuous-use intention in the context of crowdsourcing logistics involving multiple stakeholders (e.g., platform providers, merchants, free couriers, and consumers). Additionally, the study explored the moderating effect of perceived risk on the correlations between satisfaction and continuous-use intention, perceived value and continuous-use intention, and trust and continuous-use intention.

While this study provides valuable insights into consumer behavior in crowd-sourcing logistics services, several limitations should be acknowledged, offering avenues for further research. Firstly, the sample selection was limited to first and second-tier cities in China, which may not fully represent the diverse attitudes of consumers across different regions and nationalities. Future research should consider expanding the sample size and conducting cross-country comparisons to better understand variations in consumer perceptions. Additionally, the model's interpretation of continuous-use intention could be further enhanced by exploring additional factors such as social norms and perceived behavioral control. Future studies could delve deeper into these factors to improve the model's predictive power. Furthermore, the study did not consider the types of goods being delivered, which could influence the correlations among variables due to differences in delivery requirements and characteristics. Investigating the impact of goods types on consumer behavior could provide valuable insights for both academia and practitioners in the logistics industry. By addressing these limitations, future research can contribute to a more comprehensive understanding of consumer behavior in crowd-sourcing logistics services.

## Data availability statement

Data will be made available on request.

## CRediT authorship contribution statement

**Yu Liu:** Writing – original draft, Methodology, Funding acquisition. **Meng Shang:** Writing – review & editing, Supervision, Conceptualization. **Chunjie Jia:** Project administration. **Xin-Jean Lim:** Writing – review & editing, Supervision. **Ye Ye:** Validation, Writing – review & editing.

## Declaration of competing interest

The authors declare no conflict of interest.
